# Toxic Elements in Sheep Milk, Whey, and Cheese from the Environmentally Burdened Area in Eastern Slovakia and Health Risk Assessment with Different Scenarios of Their Consumption

**DOI:** 10.3390/toxics12070467

**Published:** 2024-06-27

**Authors:** Simona Almášiová, Róbert Toman, Martina Pšenková, Vladimír Tančin, Ivona Jančo

**Affiliations:** 1 Faculty of Agrobiology and Food Resources, Institute of Animal Husbandry, Slovak University of Agriculture in Nitra, Trieda Andreja Hlinku 2, 949 76 Nitra, Slovakia; xalmasiova@uniag.sk (S.A.); martina.psenkova@uniag.sk (M.P.); vladimir.tancin@uniag.sk (V.T.); 2 AgroBioTech Research Center, Slovak University of Agriculture in Nitra, Trieda Andreja Hlinku 2, 949 76 Nitra, Slovakia; ivona.janco@uniag.sk

**Keywords:** sheep milk, sheep cheese, sheep whey, toxic elements, potentially toxic elements, health risk assessment, consumption of sheep milk, Slovakia, environmental burden, target hazard quotient

## Abstract

The study aimed to determine the content of 17 elements (Al, As, Ba, Cd, Co, Cr, Cu, Fe, Li, Mn, Mo, Ni, Pb, Sb, Se, Sr, and Zn) in samples of sheep milk, cheese, and whey (36 samples in total) collected from a farm in an environmentally burdened area due to the long-term mining and industrial activity in Slovakia as well as to determine the possible risk of consumption via health risk assessment calculations. Consumption of 120 g of milk, 500 g of milk, 20 g of cheese, and 100 g of cheese were used in calculations for children and adults, respectively. According to the results, concentrations of four elements are controversial. Lead concentrations in all types of samples exceeded the maximum permissible lead limit in milk set by European Union legislation. The content of Se and As is problematic for children’s consumption, and the target hazard quotient for As and Al is higher than one (considered potentially not safe) in all scenarios. According to the target system approach, lead concentrations in milk and cheese could adversely influence the nervous system and kidneys of adults’ and children’s developmental and reproductive systems. Considering the worst-case scenario, consuming sheep milk and cheese from the monitored areas could represent a risk and be potentially harmful to human health, mainly for children. However, further monitoring of the levels of elements and concentrations in environmentally burdened areas and more robust data on consumption are needed.

## 1. Introduction

Numerous studies confirm that consuming milk and dairy products contributes significantly to human health on several levels, supports the proper functioning of the body organs, and is part of preventing various diseases that can be influenced by diet [1,2,3,4]. Authorities recommend consuming two to three portions of milk and dairy products daily, mainly for children who belong to risky groups, to ensure that the nutritional requirements for proper development and growth are met [3,5]. According to Haug et al. [2], daily consumption of 0.5 L of milk fulfills many of the nutrients required daily for humans.

Sheep milk is considered more digestible and is better tolerated in allergic individuals and individuals with certain skin and respiratory diseases. Consumption of sheep milk and dairy is recommended as part of a therapeutic diet in clinical and medical nutrition [6]. Kawecka and Pasternak [7] stated that people can benefit greatly from goat and sheep milk, and cheese made from sheep milk is highly valued because of its nutritional properties, the tradition of its production, and palatability. Milk and dairy products may also contain various amounts of contaminants, such as pesticides or heavy metals residues, which are widely distributed in the environment. Toxic elements transferred through the food chain may harm human health and cause adverse effects [8]. According to the definition from FAO/WHO [5], adverse (health) effects represent a change in the morphology, physiology, growth, development, reproduction, or life span of an organism, system, or (sub)population that results in an impairment of their functional capacity, an impairment of their capacity to compensate for additional stress, or an increase in their susceptibility to other influences. Even small amounts of toxic elements can pose adverse health effects upon regular consumption [9], and the intake of toxic elements represents a higher risk for vulnerable groups, such as children, the elderly, and people with diseases [8].

On the one hand, a higher intake of milk and dairy products is recommended for children, but children are more sensitive to the cumulative effects of toxic elements. Accumulation of potentially present toxic elements in food can occur at any age, but the highest predisposition to accumulation concerns children during their first 13 years of life [10]. The reason is that during an organism’s development, children consume more food per unit of body weight than adults [8]. The second reason is that the body of a child is more sensitive due to the development and growth stage [8,9]. Therefore, it is crucial to monitor the level of trace elements in milk and dairy products, which are significant sources of nutrition, particularly in childhood. The presence of toxic elements in milk is controlled according to the EU’s defined maximum level by European regulation No. 1881/2006 [11].

Monitoring of the content of toxic elements in environmentally stressed areas due to mining and subsequent processing activities can be observed in many global and European studies. On the territory of the Slovak Republic, one such area is Stredný Spiš, an example of an environmentally burdened area due to long-term mining and industrial activity, which has already ended [12]. Even now, thirty years after the end of mining, we can still perceive the negative consequences of mining and raw material processing in the mentioned area, as well as around the monitored farm. Extraction of mineral raw materials has left irreversible changes underground and on the surface (mined areas, collapse, heaps, and tailing ponds), but there are serious concerns of contamination of all components of the environment with heavy metals, as well [12,13,14]. The surroundings of the monitored farm were contaminated mainly with As, Cu, and Zn [14]. Although mining activities were stopped or at least significantly limited at the beginning of the 21st century, the presence of heavy metals in soils has not decreased due to heavy metals’ persistence and non-biodegradability [13]. However, polluted soils become a source of contamination for the food chain [15]. There is a long tradition of sheep dairy products being consumed in the region where the monitored farm is located. Dairy farming, a creamery, and a company store are also situated close to the farm. Products made there are distributed throughout the country. However, data on exposure levels of toxic elements through food sources are currently missing since monitoring studies have not been conducted. The present study aimed to determine the content of toxic and essential elements, which are considered as potentially toxic elements, in samples of sheep milk and sheep dairy products collected from a farm in a polluted area in Slovakia to monitor and compare the current occurrence of elements in the first step and to refer to the suitability of the usage for human consumption according to results of the risk assessment in the second. Health risk assessment is a process intended to calculate or estimate the risk to a given population, including identifying attendant uncertainties regarding exposure to a particular agent, which is an integral part of food safety policy [16,17].

## 2. Materials and Methods

### 2.1. Collections of Samples and Characteristics of a Region

**Figure 1:**
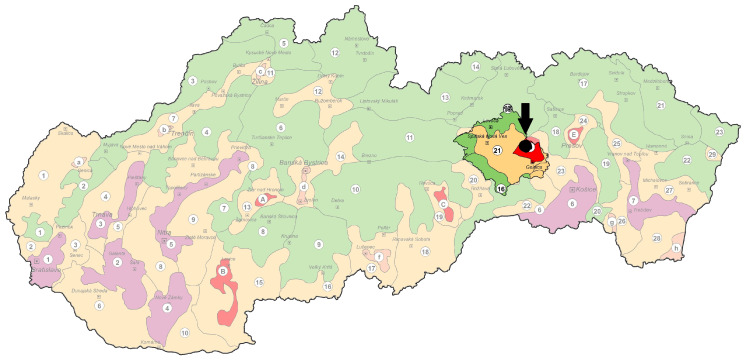
Environmental regional classification of Slovakia with marked monitored region [19]. Note: The approximate location of the farm from which samples were taken is marked with a black circle. Region Spiš is highlighted with a brighter color. Legend: green-colored areas = regions with an undisturbed environment, yellow-colored areas = regions with moderately disturbed areas, and red- and purple-colored areas = regions with highly and strongly disturbed environments.

Samples of sheep milk, whey, and cheese from the selected farm were collected. The farm (approximate location is shown in Figure 1) is located in a region that is considered to belong to heavily disturbed areas according to the division made by The Ministry of the Slovak Republic and the Slovak Environmental Agency. The authorities have determined that the environmental regionalization of Slovakia and the country is divided into three types of environmental quality: regions with potentially undisturbed areas, regions with slightly disturbed areas, and regions with heavily disturbed areas [18]. The region, called Spiš, is located in the Eastern part of the country and contains areas of all three quality types. The area with a heavily disturbed environment represents a relatively small part of the region (Figure 1). The specific characteristics of the heavily disturbed area of the region explained below represent one of the reasons why the samples were taken at this particular farm. To characterize the surroundings of the farm from which we took the samples used in this study, we need to mention the metallurgical factory, explicitly specialized in the production of refined copper, which is located nearby, and we can assume that it may have a significant impact on the quality of the environment. The region is known for its mining history, as well. Despite the termination of mining activities, increased concentrations of heavy metals are still present. The area still has rich reserves of mineral raw materials, especially copper and iron ore. Several hundred years of mining and metalworking together with its volcanic origin predispose the region Spiš to higher levels of contamination by heavy metals and metalloids such as As, Cd, Cu, Fe, Hg, Mn, Pb, and Zn. As a result of mining, tail pods and dumps of the mined and processed polymetallic ores represent a secondary source of contamination [12,14]. More geomorphological specification of the region is written in the study by Árvay et al. [14].

We took samples of sheep milk, whey, and fresh cheese during the lactation period every two weeks. From April to late September, 12 samples of each product were collected, 36 samples in total. The ecological farming method is used on the farm, and the herd consists of approximately 450 ewes of the improved Valachian breed x Lacaune. At the same time, dairy products (whey and cheese) are made exclusively from fresh milk in selected farm in areas with heavily disturbed environments. In other local production places, mixed milk from more farms and even more countries is used for dairy production.

### 2.2. Samples Preparation

Samples of sheep milk were taken from milk tanks to represent all individuals and stored in freezers at −18 °C until analysis was carried out. For the production of whey and cheese, only milk originally from the selected farm was used, and additional milk was mixed during preparation. Samples of whey were taken from the container, and samples of cheese were cut from a representative bigger cheese and consequently stored in the same way as the samples of milk. A preanalytical procedure such as homogenization was performed first in all samples. The weight of the experimental samples ranged from 1.0 to 2.0 g and was reflected in measurement. Sampling and storage steps were optimized to reduce all possible contaminations, losses, or alterations that could negatively affect data reliability. Only plastic tools for handling and tubes for storage were used during preparation. The homogenizer Heidolph DIAX 600 (Heidolph Elektro GmbH & Co. KG, Schwabach, Germany) was used for homogenization.

### 2.3. Analysis of Samples

The occurrence of 17 chemical elements (Al, As, Ba, Cd, Co, Cr, Cu, Fe, Li, Mn, Mo, Ni, Pb, Sb, Se, Sr, and Zn) which may cause harmful effects on human health was analyzed by the following procedure. Analysis of the elements was determined using inductively coupled plasma–optical emission spectrometry (ICP OES) with axial plasma configuration and with auto-sampler SPS-3. As the first step, samples were mineralized in the high-performance microwave digestion system Ethos UP (Milestone Srl, Sorisole, BG, Italy) in a solution of 5 mL HNO₃ ≥ 69.0% (TraceSELECT^®^, Honeywell Fluka, Morris Plains, NJ, USA). A total of 1 mL H₂O₂ ≥ 30% was used for trace analysis (Sigma Aldrich, Saint–Louis, MO, USA) and 2 mL of ultrapure water 18.2 MΩ cm^−1^, 25 °C (Synergy UV, Merck Millipore, France). The method of determination consisted of heating and cooling phases. Consequently, analysis of the elements was carried out using an inductively coupled plasma–optical emission spectrometer (ICP OES 720, Agilent Technologies Australia (M) Pty Ltd., Mulgrave, VIC, Australia) with axial plasma configuration and with auto140 sampler SPS-3 (Agilent Technologies, Mulgrave, VIC, Australia). Detection limits (mg kg^−1^) of measured trace elements were as follows: Al 0.0002; As 0.0015; Ba 0.00003; Cd 0.00005; Co 0.0002; Cr 0.00015; Cu 0.0003; Fe 0.0001; Li 0.00006; Mn 0.00003; Mo 0.0005; Ni 0.0003; Pb 0.0008; Sb 0.002; Se 2.0; Sr 0.00001; and Zn 0.0002; and wavelengths of determination (nm) were as follows: Al 167.019; As 188.980; Ba 455.403; Cd 226.502; Co 228.615; Cr 267.716; Cu 324.754; Fe 234.350; Li 670.783; Mn 257.610; Mo 204.598; Ni 231.604; Pb 220.353; Sb 206.834; Se 196.026; Sr 407.771; and Zn 206.200. The legitimacy of the whole method was verified using the certified reference material (CRM: Yttrium (CAS n.: 1314-36-9), Indium (CAS n.: 7440-74-6), Rhodium (CAS n.: 10049-07-7) ERM: BD 151, BB 184BCR 274). Details of the instrumental operating conditions are listed in Table 1.

### 2.4. Statistical Analysis

All results of this study were processed using Statistica Cz version 10 (TIBCO Software, Inc., Palo Alto, CA, USA). All obtained results are listed as mean values with standard deviation. The median is stated in separate columns, as well. Differences in concentrations of the analyzed elements in sheep milk, whey, and cheese were compared by the ANOVA and Sheffe’s *t*-test. mA probability levels of *p* < 0.05. *p* < 0.01 and *p* < 0.001 were considered statistically significant.

### 2.5. Human Health Risk Assessment

Health risk assessments were calculated to determine the possible risk of humans consuming sheep milk and dairy products. The consumption of sheep milk (as a beverage) is not so typical currently in Slovakia. Still, calculations for risk assessment have been made since sheep milk is an important ingredient for further food production, which is not the case for sheep whey. Slovakia has no data about whey consumption, so its health risk assessments have not been calculated. On the other hand, sheep cheese consumption is rising, especially when bought locally from small farmers [20,21].

Health risk assessment of toxic and potentially toxic elements present in milk samples was made for two age groups, adults and preschool children, who already consume milk and dairy products and represent a risk group of consumers [8,9,10].

Calculations were made with two different amounts of consumption. In the first case, data from the Statistical Office of Slovakia were used, and in the second scenario, the amount of the consumed portion was increased to the recommended nutritional level.

The first step of health risk assessment, the calculation of EDI (estimated daily intake), was made using the following formula recommended by different authors [22,23,24].

$$EDI=\frac{\left(C\times W\right)}{BW}$$

where *C* (mg/kg) represents the mean level of the monitored element, *W* represents daily milk consumption in Slovakia, and BW represents body weight. For an adult person, 70 kg was used in calculations. In the case of children, we also used data from the EFSA Scientific Committee [25], where a weight of 22 kg for children (3–10 years) is listed. According to the Statistical Office of Slovakia, the mean daily milk consumption in Slovakia is 120 g per person, and the mean daily consumption of fresh cheese is 20 g per person [26]. More precise data from food frequency questionnaires, which exist for some European countries and are possibly found in the FoodEx2 tool from EFSA (Parma, Italy), are currently missing for the Slovak Republic. The collection of proper data is ongoing by the national EFSA office. So, for the second scenario, we set the following portions: 500 g of milk and 100 g of cheese consumed daily.

Since for toxic metals Al and As only EWI (estimated weekly intake) exists, they were calculated by the equation [27,28]:

EWI=EDI×7

Consequently, for percentage expression of risk, %TDI (tolerable daily intake) was expressed for toxic elements Ba, Ni, Sb, and Sr; %PTWI (provisional tolerable weekly intake) for Al and As; and %p-RfD (provisional oral reference dose) for Li [29].

For potentially toxic elements—Cu, Fe, Se, and Zn—essential for the human body [30,31], we computed the recommended doses for the Slovak population [32]. Concentrations were compared with values for preschool children (4–6 years old) and adult groups with the highest needs (Cu and Zn for men with physically hard work, Fe for pregnant women in the second trimester, Se for breastfeeding women). Concentrations for Mn were compared with the values for children and adults that are used as RDAs (Recommended Dietary Allowances), which include 1.5 mg for children and 2.3 mg for adults [33]. If an essential element exceeded the fulfillment of its required intake and a tolerable upper intake (UL) was set for this element, a calculation was also made using this limit to assess the risk of intake of this element.

The target hazard quotient (THQ) and hazard index determination (HI) were calculated in the present study. The US Environmental Protection Agency established THQ to estimate the non-carcinogenic risk associated with the reference dose and exposure [34]. For calculation, the following formula was used [24]:

$$THQ=\frac{EDI}{RfDo}$$

where *EDI* is already explained, and *RfDo* is the reference oral dose (mg/kg/day). The reference doses for aluminum, arsenic, barium, cadmium, chromium, lithium, molybdenum, nickel, lead, antimony, strontium, zinc, copper, and iron are 0.0004, 0.0003, 0.2, 0.001, 0.003, 0.002, 0.005, 0.02, 0.0035, 0.0004, 0.6, 0.3, 0.04, and 0.7 mg/kg of body weight per day, respectively [34,35,36]. Potential non-carcinogenic effects may occur if the THQ value is greater than 1. Adverse health effects are unlikely to be caused when THQ is less than 1 [24]. Typically, we are exposed to mixtures of contaminants in low doses instead of a single contaminant, and to assess the cumulative risk of multiple metals, a hazard index was performed by summing the THQ of each metal monitored in the present study. HI value below 1 means that sheep milk or cheese consumption is safe, whereas a hazard index higher than 1 indicates that long-term and frequent consumption could be associated with potential adverse health consequences that may occur over time [22,24]. For human health, animal health, and ecological mixture risk assessments, the EFSA is collecting available knowledge on methodologies [37]. Using the principle of dose addition, they present a tiered approach to component-based mixture risk assessment. The HI calculation sums all hazard quotients regardless of the toxicological endpoint for all components. A refined approach is considered as the HI approach develops since not all components have the same adverse effects/target organs. Target organ toxicity doses are calculated for each endpoint to estimate an endpoint-specific HI. Boberg et al. [38] presented a pragmatic step-by-step procedure for mixture risk assessment, and the authors proposed tools for grouping chemicals, which we used for further, advanced calculations of health risk assessment of milk and cheese consumption from the monitored heavily disturbed region. These approaches require a high level of information about chemical exposures and toxicities, which is often lacking. We could only calculate Al, Cd, Pb, and Ni since data were available just for those elements [38]. In the present study, we used toxicity data related to each cumulative assessment group and derived tolerable dose (DTD) for the mentioned elements (Al, Cd, Pb, and Ni) from the tool chemicalmixturecalculator.dk [39]. The calculation of THQ is the same, with the difference being that the DTD value is entered into the formula instead of RfDo (the reference oral dose). Afterward, the HI is added together for cumulative target groups [40]. 

## 3. Results and Discussion

Regarding nutrition, the elemental content in milk and dairy products can be divided into essential and nonessential content [40]. The amounts of toxic elements usually are minimal in pure milk and dairy products. Many hazardous elements or compounds accumulate along the food chain due to increased urban, agricultural, and industrial emissions. The extent of metal toxicity is closely related to age, sex, the route of exposure, the amount of the dose, solubility, the oxidation state of the metal, the retention percentage, the duration of exposure, the frequency of intake, the rate of absorption, and the effective rejection mechanism [41,42]. In addition, it has been reported that heavy metals can contaminate raw milk through the machinery and equipment used in milk processing and distribution [43]. Levels of essential elements, which can be potentially harmful when overdosed (Cu, Fe, Mn, Se, and Zn), and toxic elements (Al, As, Ba, Cd, Co, Cr, Li, Mo, Ni, Pb, Sb, and Sr) in sheep milk, whey, and cheese are listed in Table 2. The mean concentrations were found and are listed in decreasing order as follows: Zn > Fe > Sr > Al > As > Ba > Se > Cu > Ni > Sb > Mn = Li > Pb > Mo > Cr = Co in milk, Fe > Zn > As > Se > Sr = Ni > Al > Cu > Cr= Li > Pb > Mn = Co > Ba > Sb > Mo in whey, and Zn > Fe > Sr >Al > Ba > As > Ni > Se > Mn > Cu > Sb > Pb > Li > Mo > Cr > Co in cheese. Cadmium was not detected in any sample from the selected farm with an ecological way of farming. Moreover, organic dairy farms are characterized by lower concentrations of toxic elements in whole raw milk than those from the conventional production system [44]. To compare with results from previous studies by our research team, we can state that there are higher concentrations of risk and toxic elements in samples from the monitored areas. In contrast, the same elements were not detected in other regions (mainly regions representing potentially undisturbed or slightly disturbed areas) [44,45,46,47,48,49,50]. The only exceptions are results from the comparative study of element concentrations in sheep milk from farms from three different regions, of which one farm is located in the region Spiš, in the part of the region that represents a heavily disturbed area as well, and the farm is situated closely to the farm our samples came from. Levels of many elements (for example, Al, As, Ba, Cu, Fe, Li, Mn, Sb, Se, and Zn) from a farm in the previous study agreed with the results of the presented research, except for the concentration of lead. In our case, the concentration of Pb in milk is two times higher [45]. For Al, Ba, Fe, Mn, Mo, Ni, and Zn, the concentrations of elements are statistically higher (* *p* < 0.05; ** *p* < 0.01; *** *p* <0.001) in cheese. Cheese is a nutrient-dense food, considered to be a concentrate of milk compounds [21,51].

We used the data from Table 2 to evaluate individual elements through calculations of risk assessment. Table 3 lists the estimated intakes of elements, applicable limits, and % of applicable limits for particular elements in the case of adults. In Table 4, the same data regarding children’s consumption are listed. In Table 5, we state the estimated intakes for potentially toxic elements that are essential and the fulfillment of recommended daily intakes.

### 3.1. Cadmium

According to the Opinion of the Scientific Panel on Contaminants in the Food Chain [59], the contamination of animal feed by cadmium cannot be entirely avoided, given its ongoing occurrence in the environment. However, according to the results of the analyses, we could not detect the presence of cadmium in our samples. The concentration of cadmium in sheep milk and dairy products is lower than the limit of detection (0.00005 mg kg^−1^), which is a favorable finding since cadmium as a food-chain contaminant represents a significant health hazard [60]. The biomonitoring study “Democophes” showed that Slovakia and Poland had the highest cadmium exposure in mothers and children [61]. We can assume (according to scientific evidence as well as the fact that permissible limits of Cd in milk and dairy products were not set by the European Commission [11]) that sheep milk is not a significant source of dietary Cd; however, since food is the primary exposure in the non-smoking population, Cd levels in food products should be kept as low as possible [61]. However, Totan and Filazi [62] stated that high levels of cadmium and lead have been found in many studies that monitor the content of toxic elements in dairy products. These elements are often found in samples from areas with higher industrialization and related activities nearby [63].

### 3.2. Lead

Lead and cadmium are considered the most widespread xenobiotic metals in the environment and, simultaneously, the most dangerous for human consumption [64]. There is evidence that even regular and long-term consumption of low amounts of Pb can pose health problems, as lead is highly cumulative, causes pathological changes, and is carcinogenic [65,66,67]. According to the European Commission [11] and Codex Alimentarius [68], the maximum permissible value for lead in milk is 0.020 mg kg^−1^. The mean concentration of Pb in samples from the Spiš region was 0.04 mg kg^−1^ in milk, 0.09 mg kg^−1^ in whey, and 0.13 mg kg^−1^ in cheese. The median values were slightly lower but higher than the set maximum permissible limit. The allowable limit was exceeded in 75% of milk samples, 58% of whey samples, and 92% of cheese samples. Many studies have detected a higher lead content in milk than the set limit [69,70,71]. In previous studies, Toman et al. [50] also determined an above-limit lead concentration in the Orava region (Slovakia). However, in other studies from Orava, the lead content was below the detection limit [48,72]. A Provisional Tolerable Weekly Intake (PTWI) of 0.025 mg kg^−1^ for lead has been withdrawn because, according to WHO [56] and EFSA [73], it is not possible to determine a tolerable intake that is not harmful to human health. Since the limit was withdrawn, there is no point in counting the % PTWI. Just for comparison, a report from the SCOOP 3.2.11 project in 2004 stated that the weekly %PTWI for the mean adult population represented 0.02–0.78% of the PTWI from milk and dairy products across the participating European countries. However, lead concentrations in milk and dairy products were lower than the permissible limit [74].

### 3.3. Arsenic

The concentration of As in milk was 0.46 mg kg^−1^; in whey, 0.24 mg kg^−1^; and in cheese, 0,73 mg kg^−1^. The CONTAM Panel [75] recommended reducing dietary exposure to inorganic arsenic. A PTWI has been established for arsenic in drinking water (0.015 mg kg^−1^ bodyweight) but not in the form of inorganic arsenic and not for other foodstuffs, and no recommendations are presently given for the intake of arsenic by children [28]. There currently is no legislation in force for arsenic in foodstuffs within the EU, except rice and rice products [11]. It is essential to state that the mentioned facts can be potential sources of uncertainty when determining conclusions. However, according to Table 3 and Table 4, determined As concentrations represent 9.7–153% PTWI for adults and 30.9–487% PTWI for children. According to the European project SCOOP 3.2.11 report, only seafood significantly impacts the intake of arsenic, and the mean weekly intake of As through milk is 0.009 mg kg^−1^ for the mean adult population. Arsenic in dairy represents <1–6.8% of the total dietary intake of As [74].

### 3.4. Aluminium

Aluminum concentrations were measured in milk, whey, and cheese, resulting in 0.53 mg kg^−1^, 0.13 mg kg^−1^, and 3.35 mg kg^−1^, respectively. According to calculations, these concentrations represent 0.3–1.7% PTWI for adults and 1–5.3% PTWI for children. Aluminum enters the milk and milk products from a variety of sources. One of them is contamination from the metal processing equipment during production. Using aluminum utensils for processing and storing milk may substantially increase the level of this metal in milk and milk products. The levels of aluminum in a study from Egypt were 19.93 ppm in farm milk, 107.32 ppm in market milk, 52.34 ppm in Kareish cheese, 4.19 ppm in yogurt, and 80.97 ppm in rice pudding. Which represent daily intakes of aluminum from farm milk, market milk, Kareish cheese, yogurt, and rice pudding, which are 3.99, 21.46, 1.15, 0.06, and 8.6 mg/day, respectively. These values are equivalent to 23.25, 125.21, 6.72, 0.35, and 50.1% of the PTWI [40]. Long-term exposure to aluminum causes neurotoxicity, brain aging, and some neurodegenerative diseases such as Parkinson’s disease [76].

### 3.5. Strontium

The detected strontium concentrations were 1.04 mg kg^−1^ in milk, 0.17 mg kg^−1^ in whey, and 4.96 mg kg^−1^ in cheese. For the presence of strontium in samples from the region Spiš, it is recommended one take into account the sufficient consumption of calcium. Since the present level of strontium in samples represents only 5% of the contribution of the total daily intake for strontium for an adult man/woman in case of a higher consumed portion of milk and cheese, for a child, it is 18% from TDI for milk and 17% for cheese. The amount is still safe, but this element is an antagonist of calcium; it adheres to the surface of bones in adulthood, and during childhood, it can be used to form the mineral part of the bone, and thus, it is stored in the body for many years. If there is a lack of calcium and protein in a child’s diet, strontium can cause poor bone growth [77].

### 3.6. Copper

The detected copper levels in milk, whey, and cheese were 0.14 mg kg^−1^, 0.12 mg kg^−1,^ and 0.26 mg kg^−1^, respectively, representing 0.3–16% of the recommended daily intake. According to Bigucu et al. [78], daily consumption of 500 g of milk and dairy products will ensure approximately 0.35 mg, which is higher than our results. Copper is an essential micronutrient and regulated product used in organic and conventional farming pest management. Both deficiency and excessive exposure to copper can have adverse health effects [78].

### 3.7. Zinc

The zinc concentrations were 4.69 mg kg^−1^ in milk, 0.25 mg kg^−1^ in whey, and 24.29 mg kg^−1^ in cheese. The examined amounts represent 3–75.8% of the daily recommended intake of Zn for adults (men with physical hard work, 16 mg) and 11.26–242.80% of the daily recommended intake of Zn for preschool children (5 mg) [32]. A higher intake of sheep cheese (100 g) provides the highest fulfillment of daily needs. It has been found that almost 99% of zinc in milk is found in skim milk, primarily in casein micelles. Hard cheeses are a good source, as their zinc concentration is over 20 mg kg^−1^. Regarding the daily need for zinc (12–14 mg), two glasses of milk provide up to 20% of the recommended dose [79]. Monitored sheep cheese appears to be a significant source of zinc.

### 3.8. Selenium

The analysis revealed that the selenium concentration in milk was recorded at 0.21 mg kg^−1^, while whey and cheese exhibited concentrations of 0.22 mg kg^−1^ and 0.38 mg kg^−1^, respectively. According to calculations of the fulfillment of daily needs of Se established by the Slovak Ministry of Health (0.025 mg for children/0.075 mg for breastfeeding women, the population group with the highest need for Se) [32], milk and cheese contain the amount of Se which fulfills this need to 101% (120 g of milk), 420% (500 g of milk), 30% (20 g cheese), and 760% (100 g of cheese) for preschool children and 33% (120 g of milk), 140% (500 g of milk), 10% (20 g of cheese), and 253% (100 g of cheese) for adults, breastfeeding women, specifically. The tolerable upper intake level (UL) of Se was set last year by the EFSA [80] as 0.095 mg/day for children 4–6 years old and 0.255 mg/day for adults, including lactating women. This means that, theoretically, the consumption of 500 g of milk by a child would exceed the established UL for the intake of Se. If a child consumed 100 g of cheese, the UL would be exceeded twice. In the case of all consumption scenarios for adults, the Se intake is lower than the established upper limit. Alopecia, as an early observable feature and a well-established adverse effect of excess selenium exposure, is selected as the critical endpoint on which the base of the UL for selenium was set [80]. In the EFSA’s intake assessment of selenium in European populations, the main food groups contributing to selenium intake were milk and dairy products, meat and meat products, grains, and fish [81].

### 3.9. Manganese

The concentration of Mn was 0.06 mg kg^−1^ in milk, 0.01 mg kg^−1^ in whey, and 0.30 mg kg^−1^ in cheese. The detected concentrations represent 2–10% fulfillment of the daily recommended intake of Mn for children and 1.3–6% for adults with higher consumed portions. According to the EFSA [82], the safe level of Mn intake is 4 mg/day in the case of preschool children and 8 mg/day in the case of adults, and an upper limit for manganese is possible to establish for any population group right now. However, individuals with impaired hepatic function, iron deficiency, or specific genetic mutations could be vulnerable and at higher risk of manganese toxicity. Absorption of manganese in the intestine is relatively low, less than 10%, which appears to be an adaptive regulation allowing manganese homeostasis to be maintained [83].

### 3.10. Nickel

The respiratory and immunological systems are the most sensitive to nickel toxicity following inhalation or oral exposure. [55]. After absorption, nickel is widely distributed in an organism [84]. The analysis revealed that nickel concentrations in milk were recorded at 0.09 mg kg^−1^. In comparison, whey and cheese exhibited concentrations of 0.17 mg·kg^−1^ and 0.70 mg kg^−1^, respectively, which means almost 4–24% TDI for children and 1.2–7.7% for adults.

### 3.11. Iron

The risk of systemic iron overload from dietary sources is negligible, with normal intestinal function [33]. Moreover, milk and dairy products are not considered a significant source of iron [85]. Iron concentrations were 2.0 mg kg^−1^ in milk, 1.36 mg kg^−1^ in whey, and 6.57 mg kg^−1^ in cheese. However, the concentrations mentioned are higher than those in studies by other authors [42,86,87,88]. While the content of Fe in cow and goat milk is similar, iron concentrations are the highest in sheep milk [89].

### 3.12. Barium

Barium is a physiological antagonist of potassium. In foods of animal origin, barium concentrations are relatively low [90]. Concentrations of barium were measured in milk, 0.3 mg kg^−1^; in whey, 0.06 mg kg^−1^; and in cheese, 1.26 mg kg^−1^. The total daily intake of 0.2 mg·kg^−1^ of body weight was chosen as an indicative value for health to characterize the danger of barium [52]. The dose at which adverse effects could be expected has not been established. Pearson and Ashmor [91] stated that barium is not considered an element that affects human health through the food chain due to the conservatism in setting this value.

### 3.13. Other Elements—Cr, Co, Li, Mo, and Sb

Chromium, cobalt, lithium, molybdenum, and antimony are elements we found in the lowest amounts in all our samples. Their concentrations are as follows: 0.01 mg kg^−1^, 0.01 mg kg^−1^, 0.06 mg kg^−1^, 0.02 mg kg^−1^, and 0.07 mg kg^−1^ in milk; 0.09 mg kg^−1^, 0.08 mg kg^−1^, 1.36 mg kg^−1^, 0.01 mg kg^−1^, and 0.04 mg kg^−1^ in whey; and 0.04 mg kg^−1^, 0.03 mg kg^−1^, 0.07 mg kg^−1^, 0.06 mg kg^−1^, and 0.22 mg kg^−1^ in cheese, respectively. These elements are usually not associated with concerns about contaminating milk and causing adverse health effects. Their concentrations were very often lower than the limit of detection in our previous studies [44,45,46,47,48,49,50]. In a recent study from Croatia, 0.028 mg kg^−1^ Mo and 0.07859 mg kg^−1^ Cr in Lacaune sheep milk were found [92]. The limits for Co and Mo have not established TDI or PTWI limits. For Cr, the % TDI was lower than 0.5% in every calculated scenario. In a study by Wang et al. [93], chromium levels in cow’s milk demonstrated a stable range around 0.01 mg kg^−1^, which follows our results on milk. In the regions Calabria and Campania in Italy, higher concentrations of Cr were found in sheep milk (0.14 mg kg^−1^), ricotta cheese (0.32 mg kg^−1^), and fresh cheese (0.46 mg kg^−1^) [94]. A higher concentration of Cr in sheep milk (0.29 mg kg^−1^) was also found in Hungary [95]. In the case of Li, p-RfD was set as 0.002 mg·kg^−1^ [29], and concentrations of Li in our samples represent 0.1–2.1% for adults and 0.3–6.8% for children. The hazard characterization of lithium is less precise as there is ongoing consideration that lithium plays an essential role in the diet [91,96]. We found two-times-higher concentrations of Li in our samples of milk than those in samples of fermented milk in Poland, and our concentrations in whey and cheese samples agree with concentrations of lithium in bio-yogurts and yogurts [96]. The TDI for Sb is 0.006 mg kg^−1^ [57], and the % TDI varies from 0.33 to 2.6% for children and 0.1–0.8% for adults. Potential sources of antimony in food can be migration of the residual antimony catalyst in polyethylene terephthalate (PET) plastic bottles [91]. Extraordinarily high Sb content was found in a milk sample in Turkey, and the study’s authors assume the manipulation of the sample was inappropriate during the milk’s storage [43].

### 3.14. Target Hazard Quotient and Hazard Index

In addition to the calculations of %PTDI and %PTWI and other % of established limits, we calculated the total hazard quotient (THQ) and the hazard index (HI) for the toxic elements. The risk quotients for each element in different scenarios are shown in Table 6. THQ and HI make it possible to evaluate the non-carcinogenic risk for the human body caused by milk consumption [97]. Based on the calculations, we conclude that the THQ for most elements (Ba, Cr, Li, Mo, Ni, Pb, Sr, Zn, Cu, and Fe) in all used scenarios are lower than one, which means that they do not represent a risk for human consumption. In the case of Sb, the THQ is higher than one only in the case of higher consumptions of milk (THQ 3.98) and cheese (THQ 2.50) for children and higher consumptions of milk (THQ 1.25) for adults. However, the THQ is much higher for Al and As in all scenarios; they vary from 2.27 to 38.07 for Al and from 2.21 to 34.85 for As. The highest THQs of Al and As belong to scenarios related to children’s consumption. Al and As contribute the most to the HI, which, for children, is 16.84 for the consumption of 120 g of milk, 70.72 for the consumption of 500 g of, 10.53 for the consumption of 20 g of cheese, and 52.27 for the consumption of 0.1 kg of cheese. In the case of adults, the values of HI are as follows: 5.29, 22.05, 3.30, and 16.55 for the consumption of 120 g and 500 g of milk and 20 g and 100 g of cheese daily, respectively. A risk index higher than one is associated with the potential for adverse non-carcinogenic health effects on the body in case of regular and long-term raw material consumption. Regular consumption of sheep milk and cheeses, used in the presented study, according to the calculated hazard index, cannot be considered safe for adults and certainly not for children, even in the case of consuming a lower portion that was taken from the Statistical Office of Slovakia and represents the mean daily consumption of milk and cheese in Slovakia. The HI results in Algeria established by Boudebbouz et al. [24] for three age groups and three cow’s milk intake scenarios (one, two, and three servings per day) are as follows: 1.32–3.98 for adults, 3.10–9.30 for children, and 9.30–27.89 for infants. On the contrary, Hasan et al. [98] indicated the safety of fresh and pasteurized milk consumption for children and adults in Bangladesh based on HI values significantly less than one (0.0343, 0.221 for children; 0.089, 0.176 for adults). Based on data on blue cheese consumption (Roquefort, Danable, Gorgonzola, and Blues Stilton) from several countries (Denmark, Spain, France, Italy, and England), Reinholds et al. [99] calculated a risk index in the range of 0.00–0.14 for several age categories of the given population.

Table 7 shows that milk and cheese consumption (only in a scenario when 100 g of cheese is consumed) by adults could be problematic and cause adverse health effects influencing the nervous system and kidney health. In the case of children’s consumption, consumption of both milk and cheese could negatively affect the nervous system of children. Risk is decreasing in the following order: consumption of 500 g sheep milk, 100 g sheep cheese, 120 g sheep milk, and 20 g sheep cheese. A tolerable dose for Pb effects on kidneys during childhood was not given. However, consuming 500 g of sheep milk and almost 100 g of sheep cheese could negatively affect children’s developmental and reproductive systems. Using this approach, we can obtain a conservative (cautious) starting point for overall conclusions, which enhances chemical risk assessment and implies some uncertainty in the evaluations. Boberg et al. [38] acknowledged that HI is not always a reliable indicator of risk since it relies on hazard and exposure values that can be uncertain.

## 4. Conclusions

Concentrations of toxic elements found in sheep milk, whey, and cheese indicate that they come from a polluted environment, which indirectly confirms the classification of the monitored part of the region Spiš as a heavily disturbed area by the Ministry of the Slovak Republic and the Slovak Environmental Agency. The presence of sixteen elements was detected, and the concentration of Cd was lower than the LOD. Concentrations of lead in all types of samples exceeded the maximum permissible limit of lead in milk (0.02 mg kg^−1^), set by EC Regulation No. 1881/2006 and Codex Alimentarius General Standard for Contaminants and Toxins in Food and Feed (Codex Stan 193-1995) [68]. From the health risk assessment, there are four fundamental findings to emphasize that the consumption of milk and cheese from the monitored region can potentially represent a hazard to human health—exceeding the upper limit of Se daily intake, exceeding the PTWI limit for As in case of children consumption, or THQ > 1 of As and Al and THQ > 1 of Pb in calculation for the target group system—for nervous system and kidney health of adults and the nervous, developmental, and reproductive systems of children. For a hazard to become a severe risk, it depends on the consumption intensity and frequency at which precise data are unavailable. Still, the pilot work has revealed a data gap that would aid in making recommendations for consumers. As a pleasant discovery of the study, we can consider sheep cheese a good Zn source.

To conclude, we are aware that consumed amounts of sheep milk, mainly, could be overestimated for the majority of the population, but it is important to emphasize that toxic elements persist during food production; thus, they are also found in dairy products, even in a more concentrated form. And since in some families, the consumption of sheep cheese can be traditional, part of the support of local production and maintaining a healthy lifestyle, an even higher daily cheese intake can be noted. The worst possible scenario and a protective approach were used in the present study, which was also due by the fact that detailed food frequency data for Slovakia are missing, as discussed above. Cheese preparation from pure milk from the monitored area is not recommended; instead, mixtures of milk should be used for the preparation of cheese within commercial production. Mixtures of milk are the primary way (but not the only way) this milk is processed. Results, therefore, have to be viewed cautiously, but they can be considered as an indicator of the quality of food raw material. This pilot study demonstrates the need for increased attention to the source since it can indicate increased risk. It is good to mention that the conclusion is restricted to the monitored farm only and cannot be generalized for whole country. Further monitoring of element levels, decreasing uncertainties, obtaining more sufficient consumption data, and providing not only health risk assessment, but rather benefit–risk assessments are recommended.

## Figures and Tables

**Table 1:** The operating parameters of determination of elements by ICP-OES.

Parameter	Value
RF Power [kW]	1.30
Plasma flow [L/min]	15.0
Auxiliary flow [L/min]	1.50
Nebulizer flow [L/min]	0.85
Replicated read time [s]	5.00
Instrument stabilization [s]	15
Sample uptake delay [s]	25
Pump rate [rpm]	15
Rinse time [s]	10

**Table 2:** Mean concentrations with standard deviation and median of monitored elements in sheep milk, whey, and cheese (mg kg^−1^).

Element	Milk		Whey		Cheese	
	$\bar{x}$ + SD	Median	$\bar{x}$ + SD	Median	$\bar{x}$ + SD	Median
Al	0.53 ± 0.56 ^a^***	0.31	0.13 ± 0.21 ^b^***	0.04	3.35 ± 2.25 ^a,b^***	2.71
As	0.46 ± 0.22	0.43	0.24 ± 0.17	0.20	0.73 ± 0.29	0.71
Ba	0.30 ± 0.14 ^a^***	0.24	0.06 ± 0.20 ^b^***	0.002	1.26 ± 0.58 ^a,b^***	1.27
Cd	ND^1^	ND^1^	ND^1^	ND^1^	ND^1^	ND^1^
Co	0.01 ± 0.01	0.01	0.08 ± 0.24	0.01	0.03 ± 0.03	0.02
Cr	0.01 ± 0.004	0.01	0.09 ± 0.28	0.004	0.04 ± 0.01	0.03
Cu	0.14 ± 0.14	0.10	0.12 ± 0.20	0.06	0.26 ± 0.08	0.23
Fe	2.00 ± 0.41 ^a^*^,b^***	1.93	1.36 ± 0.33 ^a^*^,c^***	1.41	6.57 ± 0.84 ^b^***^, c^***	6.50
Li	0.06 ± 0.03	0.06	0.09 ± 0.03	0.08	0.07 ± 0.03	0.06
Mn	0.06 ± 0.01 ^a^***	0.06	0.08 ± 0.24 ^b^***	0.003	0.30 ± 0.06 ^a^***^,b^***	0.30
Mo	0.02 ± 0.02 ^a^*	0.02	0.01 ± 0.01 ^b^*	0.01	0.06 ± 0.05 ^a,b^*	0.04
Ni	0.09 ± 0.07 ^a^**	0.06	0.17 ± 0.22 ^b^**	0.09	0.70 ± 0.52 ^a,b^**	0.56
Pb	0.04 ± 0.03	0.02	0.09 ± 0.20	0.04	0.13 ± 0.09	0,08
Sb	0.07 ± 0.05	0.07	0.04 ± 0.05	0.03	0.22 ± 0.15	0.20
Se	0.21 ± 0.24	0.13	0.22 ± 0.24	0.16	0.38 ± 0.35	0.28
Sr	1.04 ± 0.31	1.08	0.17 ± 0.10	0.15	4.96 ± 0.74	5.09
Zn	4.69 ± 1.22 ^a^***	4.89	0.25 ± 0.18 ^b^***	0.19	24.29 ± 5.60 ^a^***^,b^***	26.84

^a, b, c^ same index in the row for the same element indicates a statistically significant difference between compared regions * *p* < 0.05; ** *p* < 0.01; *** *p* < 0.001; ^1^ND = not detected, LOD = 0.00005 mg kg^−1^.

**Table 3:** Estimated daily intakes of elements based on various consumptions of milk and cheese for adults compared to TDI/PTWI/p-RfD/, which are listed in the table for the given element.

	EDI (mg/day) Milk 500 g	EDI (mg/day) Milk 120 g	EDI (mg/day) Cheese 100 g	EDI (mg/day) Cheese 20 g	Limits	% of Applicable Limit in Milk 500 g	% of Applicable Limit in Milk 120 g	% of Applicable Limit in Cheese 100 g	% of Applicable Limit inCheese 20 g
Al	0.0038	0.0009	0.0048	0.0010	2 mg·kg^−1^ PTWI [27]	1.3	0.32	1.68	0.34
As	0.0033	0.0008	0.0010	0.0002	0.015 mg·kg^−1^ PTWI [28]	153.3	36.80	48.67	9.73
Ba	0.0021	0.0005	0.0018	0.0004	0.2 mg·kg^−1^ TDI [52]	1.07	0.26	0.90	0.18
Cd	ND	ND	ND	ND	0.025 mg·kg^−1^ bw/month [53]	ND	ND	ND	ND
Co	0.0001	0.0000	0.0000	0.0000	Not Established	-	-	-	-
Cr	0.0001	0.0000	0.0001	0.0000	0.3 mg·kg^−1^ [54]	0.02	0.006	0.02	0.04
Li	0.0004	0.0001	0.0001	0.0000	0.002 mg·kg^−1^ p-RfD [29]	2.14	0.51	0.50	0.10
Mo	0.0001	0.0000	0.0001	0.0000	Not Established	-	-	-	-
Ni	0.0006	0.0002	0.0010	0.0002	0.013 mg·kg^−1^ TDI [55]	4.95	1.19	7.69	1.54
Pb	0.0003	0.0001	0.0002	0.0000	PTWI withdrawn [56]	-	-	-	-
Sb	0.0005	0.0001	0.0003	0.0001	0.006 mg·kg^−1^ TDI [57]	0.83	0.20	0.52	0.10
Sr	0.0074	0.0018	0.0071	0.0014	0.13 mg·kg^−1^ TDI [58]	5.71	1.37	5.45	1.09

ND—not detected.

**Table 4:** Estimated daily intakes of elements based on various consumptions of milk and cheese for children compared to TDI/PTWI/p-RfD/, which are listed in the table for each given element.

	EDI (mg/day) Milk 500 g	EDI (mg/day) Milk 120 g	EDI (mg/day) Cheese 100 g	EDI (mg/day) Cheese 20 g	Limits	% of Applicable Limit in Milk 500 g	% of Applicable Limit in Milk 120 g	% of Applicable Limit in Cheese 100 g	% of Applicable Limit inCheese 20 g
Al	0.0120	0.0029	0.0152	0.0030	2 mg kg^−1^ PTWI [27]	4.22	1.01	5.33	1.07
As	0.0104	0.0025	0.0033	0.0007	0.015 mg kg^−1^ PTWI [28]	487.88	117.09	154.84	30.97
Ba	0.0068	0.0016	0.0057	0.0011	0.2 mg kg^−1^ TDI [52]	3.40	0.82	2.86	0.57
Cd	ND	ND	ND	ND	0.025 mg kg^−1^ bw/month [53]	ND	ND	ND	ND
Co	0.0002	0.0001	0.0001	0.0000	Not Established	-	-	-	-
Cr	0.0002	0.0001	0.0001	0.0000	0.3 mg kg^−1^ [54]	0.08	0.02	0.06	0.01
Li	0.0014	0.0003	0.0003	0.0001	0.002 mg kg^−1^ p-RfD [29]	6.81	1.64	1.59	0.32
Mo	0.0005	0.0001	0.0003	0.0001	Not Established	-	-	-	-
Ni	0.0020	0.0005	0.0032	0.0006	0.013 mg kg^−1^ TDI [55]	15.73	3.78	24.48	4.90
Pb	0.0010	0.0002	0.0006	0.0001	PTWI withdrawn [56]	-	-	-	-
Sb	0.0016	0.0004	0.001	0.0002	0.006 mg kg^−1^ TDI [57]	2.65	0.64	1.67	0.33
Sr	0.0236	0.0057	0.0226	0.0045	0.13 mg kg^−1^ TDI [58]	18.18	4.36	17.34	3.47

ND—not detected.

**Table 5:** Estimated daily intakes of potentially toxic elements based on milk and cheese consumption for adults and children and % of daily fulfillment of listed elements from doses recommended for essential intake.

Element	Cu	Fe	Mn	Se	Zn
Recommended intake (children/group of adults with the highest need) *	800 μg/1800 μg [32]	8 mg/30 mg [32]	1.5 mg/2.3 mg [33]	25 μg/75 μg [32]	5 mg/16 mg [32]
EDI in milk 500 g	0.07	1	0.03	0.105	2.35
% contribution for children	8.75	11.11	2	420	46.90
% contribution for adults	3.88	3.33	1.30	140	14.66
EDI in milk 120 g	0.0168	0.24	0.0072	0.0252	0.5628
% contribution for children	2.1	2.66	0.48	100.80	11.26
% contribution for adults	0.93	0.8	0.31	33.60	3.52
EDI in cheese 100 g	0.13	3.29	0.15	0.19	12.14
% contribution for children	16.25	36.50	10,00	760,00	242.80
% contribution for adults	7.22	10.95	6.52	253.33	75.88
EDI in cheese 20 g	0.0052	0.1314	0.006	0.0076	0.4856
% contribution for children	0.65	1.46	0.4	30.4	9.71
% contribution for adults	0.29	0.44	0.26	10.13	3.04

* explained in the methods.

**Table 6:** Target hazard quotient for each monitored element, milk and cheese consumption amounts for children and adults, and calculated hazard index.

	Al	As	Ba	Cd	Cr	Li	Mo	Ni	Pb	Sb	Sr	Zn	Cu	Fe	HI
RfDo	0.0004	0.0003	0.2	0.001	0.003	0.002	0.005	0.02	0.0035	0.0004	0.6	0.3	0.04	0.7	
Children															
THQ Milk 500 g	30.11	34.85	0.03	0	0.08	0.68	0.09	0.10	0.26	3.98	0.04	0.36	0.08	0.06	70.72
THQ Milk 120 g	7.23	8.36	0.01	0	0.02	0.03	0.02	0.02	0.06	0.95	0.01	0.09	0.02	0.02	16.84
THQ Cheese 100 g	38.07	11.06	0.03	0	0.06	0.09	0.05	0.16	0.17	2.50	0.04	0.04	0.00	0.00	52.27
THQ Cheese 20 g	7.61	2.21	0.01	0	0.01	0.02	0.01	0.03	0.03	0.50	0.01	0.07	0.01	0.01	10.53
Adults															
THQ Milk 500 g	9.46	10.95	0.01	0	0.02	0.04	0.03	0.03	0.08	1.25	0.01	0.11	0.03	0.02	22.05
THQ Milk 120 g	2.27	2.63	0.003	0	0.01	0.01	0.01	0.01	0.02	0.30	0.003	0.03	0.01	0.005	5.29
THQ Cheese 100 g	11.96	3.48	0.01	0	0.02	0.03	0.02	0.05	0.05	0.79	0.01	0.12	0.01	0.01	16.55
THQ Cheese 20 g	2.39	0.70	0.00	0	0.004	0.01	0.003	0.01	0.01	0.16	0.00	0.01	0.00	0.00	3.30

**Table 7:** THQ and HI according to the target system approach for all milk and cheese consumption amounts for both children and adults.

Age Group	Target System	Element	Target Group	DTD *	THQ Milk 500 g	THQ Milk 120 g	THQ Cheese 100 g	THQ Cheese 20 g
Adults	Nervous system	Al	All	0.1	0.037857	0.009086	0.047857	0.009571
		Pb	All	0.00005	5.714286	1.371429	3.714286	0.742857
		HI	All		5.75214286	1.380514	3.762143	0.752429
	Liver	Ni	All	0.005	0.128571	0.030857	0.2	0.04
	Developmental and reproductive system	Al	Pregnant women	1	0.003786	0.000909	0.004786	0.000957
			men	0.27	0.014021	0.003365	0.017725	0.003545
		Pb	Pregnant women	0.0004	0.714286	0.171429	0.464286	0.092857
			men	0.0006	0.47619	0.114286	0.309524	0.061905
		Ni	Pregnant women	0.0028	0.229592	0.055102	0.357143	0.071429
			men	-				
		HI	Pregnant women		0.947663	0.227439	0.826214	0.165243
		HI	men		0.490212	0.117651	0.327249	0.06545
	Hematological system	Pb	All	0.017	0.016807	0.004034	0.010924	0.002185
	Kidney	Cd	All	0.00036	-	-	-	-
		Pb	All	0.00006	4.761905	1.142857	3.095238	0.619048
		Ni	All	0.33	0.001948	0.000468	0.00303	0.000606
		HI	All		4.76385281	1.143325	3.098268	0.619654
Children	Nervous system	Al	All	0.1	0.120455	0.028909	0.152273	0.030455
		Pb	All	0.00005	18.18182	4.363636	11.81818	2.363636
		HI	All		18.3022727	4.392545	11.97045	2.394091
	Liver	Ni	All	0.005	0.409091	0.098182	0.636364	0.127273
	Developmental and reproductive system	Al	All	-				
		Pb	All	0.0006	1.515152	0.363636	0.984848	0.19697
		Ni	All	-				
		HI	All		1.515152	0.363636	0.984848	0.19697
	Hematological system	Pb	All	0.017	0.053476	0.012834	0.034759	0.006952
	Kidney	Cd	All	-				
		Pb	All	-				
		Ni	All	0.33	0.006198	0.001488	0.009642	0.001928
		HI	All		0.006198	0.001488	0.009642	0.001928

* DTD, derived tolerable dose (mg·kg^−1^/bw/day); HI, hazard index for target cumulative group.

## Data Availability

Data will be made available on request.
